# Clinical characteristics, risk factors, immune status and prognosis of secondary infection of sepsis: a retrospective observational study

**DOI:** 10.1186/s12871-019-0849-9

**Published:** 2019-10-18

**Authors:** Yao Chen, Yanyan Hu, Jin Zhang, Yue Shen, Junling Huang, Jun Yin, Ping Wang, Ying Fan, Jianli Wang, Su Lu, Yilin Yang, Lei Yan, Keyong Li, Zhenju Song, Chaoyang Tong, Shilin Du

**Affiliations:** 1 Department of Emergency Medicine, Zhongshan Hospital, Fudan University, Shanghai, 200032 China; 2 Department of Gastroenterology, Zhongshan Hospital, Fudan University, Shanghai, 200032 China; 3 Department of Pharmacology, University of Virginia School of Medicine, Charlottesville, Virginia, 22908 USA

**Keywords:** Sepsis, Secondary infection, Immunosuppression, HLA-DR, Cytokine

## Abstract

**Background:**

Secondary infection has a higher incidence in septic patients and affects clinical outcomes. This study aims to investigate the clinical characteristics, risk factors, immune status and prognosis of secondary infection of sepsis.

**Methods:**

A four-year retrospective study was carried out in Zhongshan Hospital, Fudan University, enrolling septic patients admitted between January, 2014 and January, 2018. Clinical data were acquired from medical records. CD14^+^ monocyte human leukocyte antigen-D related (HLA-DR) expression and serum cytokines levels were measured by flow cytometry and enzyme-linked immunosorbent assay (ELISA) respectively.

**Results:**

A total of 297 septic patients were enrolled, 92 of whom developed 150 cases of secondary infections. Respiratory tract was the most common site of secondary infection (*n* = 84, 56%) and *Acinetobacter baumanii* the most commonly isolated pathogen (*n* = 40, 31%). Urinary and deep venous catheterization increased the risk of secondary infection. Lower HLA-DR expression and elevated IL-10 level were found in secondary infection group. The expected prolonged in-hospital stay owing to secondary infection was 4.63 ± 1.87 days. Secondary infection was also associated with higher in-hospital, 30-day and 90-day mortality. Kaplan-Meier survival analysis and Log-rank test revealed that secondary infection group had worse survival between day 15 and day 90.

**Conclusions:**

Urinary and deep venous catheterization increased the risk of secondary infection, in which underlying immunosuppression might also play a role. Secondary infection affected the prognosis of septic patients and prolonged in-hospital length of stay.

## Background

Sepsis accounts for a considerable number of hospital and intensive care unit (ICU) admission and adds to the overall in-hospital mortality [1, 2]. Lack of consensus and knowledge in its pathological mechanism has made the patient management difficult. With proper treatment, conditions of many septic patients became stable. However, some other patients developed secondary infection which led to the aggravation of disease and even the multiple organ dysfunction syndrome (MODS).

Previous studies have provided some findings on the risk factors of developing secondary infection, such as age, severity of primary disease, length of stay (LOS) in ICU and invasive procedures [3, 4]. Some studies also focused on the association between secondary infection and the prognosis of septic patients but the results were inconsistent in how secondary infection influenced the prognosis and whether it was the major cause of death [5, 6].

It has also been widely studied that the underlying immune dysfunction of sepsis could lead to secondary infection. The early phase of sepsis features activated inflammation process caused by systemic release of pro-inflammatory cytokines called “cytokine storm” [7, 8]. Immunosuppression is then observed at later phase of sepsis as a result of the imbalance in pro- and anti-inflammatory activities [9]. Sepsis could lead to a variety of mechanisms such as the apoptosis and autophagy of immune cells, endotoxin tolerance and relevant center nervous system regulation, which presented as immunosuppression consequently [8, 10, 11]. CD14^+^ monocyte human leukocyte antigen-D related (HLA-DR) expression is an effective biomarker of immune status, which reflects the comprehensive effect of pro- and anti-inflammatory processes during sepsis [12–14]. Low HLA-DR expression is associated with immunosuppression and higher risk of secondary infection, especially during early phase of sepsis [15–19]. Serum cytokines levels are also commonly used by clinicians to monitor immune status. A higher release of anti-inflammatory cytokines such as IL-10, together with acute pro-inflammatory activities were found in the patients prone to secondary infection [20–24].

Because of the illuminating but inconsistent findings of previous studies, clinical characteristics, risk factors and the prognosis of secondary infection of sepsis were further investigated. Additionally, the association between immune status and secondary infection of sepsis based on data of HLA-DR expression and serum cytokines levels were also explored in the current study.

## Materials and methods

### Study setting and population

A retrospective study was carried out in emergency intensive care unit (EICU) of Zhongshan Hospital, Fudan University, Shanghai, China. Patients diagnosed with sepsis on admission between January, 2014 and January, 2018 were enrolled in this study. The diagnosis of sepsis referred to The Third International Consensus Definitions for Sepsis and Septic Shock (Sepsis-3), namely suspected infection with Sequential Organ Failure Assessment (SOFA) score ≥ 2 [2]. Information of infection and SOFA score were acquired from Electronic Medical Record System (EMRS). Patients were excluded if they had one of the following conditions: ① under the age of 18; ② suffering chronic heart failure (New York Heart Function Assessment - IV), advanced malignancy, end-stage liver (Child-Pugh C) or kidney diseases (CKD-5); ③ having received in-hospital treatment in other hospitals prior to admission; ④ in-hospital LOS less than 48 h. Anti-infection treatments of included patients were applied by experienced physicians based on either etiological evidence or empirical therapy plan. The study was approved by the Ethics Committee Study Board of Zhongshan Hospital, Fudan University (record number: 2006–23).

### Diagnosis of secondary infection

Secondary infection was diagnosed according to CDC/NHSN Surveillance Definition Of Health Care-Associated Infection And Criteria For Specific Types Of Infections In The Acute Care Setting [25]. Clinical information used to identify secondary infection such as signs/symptoms and results of laboratory tests such as pathogen cultures were acquired from EMRS. Only the newly-onset nosocomial infections identified later than 48 h after admission were classified as secondary infections. The time of the onset of secondary infection was the day when positive cultures were collected, or when signs/symptoms emerged if no positive cultures were gained. Infections identified after leaving hospital were not documented. An infection caused by multiple pathogens but identified at the same time and same site was considered as one infection. Three experienced researchers were responsible for the diagnosis of secondary infection.

### Data collection

EMRS and Computerized Physician Order Entry (CPOE) were screened for available data. The following data of each patient were collected: ① baseline characteristics: age, gender, comorbidity and smoking history; ② site of primary infection; ③ index of severity of the disease on admission: Acute Physiology and Chronic Health Evaluation II (APACHE II) score, SOFA score and hemodynamic status; ④ interventions such as the use of glucocorticoids, anticoagulation therapy, mechanical ventilation, urinary catheterization, deep venous catheterization, continuous renal replacement therapy and blood transfusion (whether those interventions were applied before or after the onset of secondary infection was noticed); ⑤ occurrence time, site and pathogen of secondary infection; ⑥ LOS in hospital and ICU, the outcome of hospital stay.

### Measurement of monocyte HLA-DR expression and serum levels of cytokines

In order to explore the underlying immune mechanism of secondary infection, we acquired the data from Database of Clinical Sample and Information for Sepsis of Zhongshan Hospital, an database founded in 2008 and intended for the collection and perseveration of clinical samples of septic patients. According to the guideline of database, the peripheral blood samples were collected in the BD Vacutainer® tubes (BD Biosciences, CA, USA) at day 1, 3 and 7 after admission. In some patients, samples at day 3 and 7 were not collected due to specific clinical conditions. Thus, data of only a part of the included patients were available as the limitation of a retrospective study. To explore CD14^+^ HLA-DR^+^ monocytes expression, a following double color staining was utilized: a fluorescein conjugated (FITC)-CD14, allophycocyanin conjugated (APC)-HLA-DR (BD Biosciences, CA, USA), according to manufacturer’s instructions. Appropriate isotype controls were run with healthy controls and used for compensation and gating blood samples. Subsequently, samples were analyzed on a 18-parameter BD LSR Fortessa analyzer (BD Biosciences, CA, USA) with FlowJo software (Tree Star Inc., OR, USA). HLA-DR expression was shown as the percentage of CD14^+^ HLA-DR^+^ monocytes among all CD14^+^ monocytes. The levels of tumor necrosis factor-α (TNF-α), interleukin-6 (IL-6), IL-8 and IL-10 were measured by ELISA method (R&D System, MN, USA) according to manufacturer’s instructions. The experiments of flow cytometry and ELISA were conducted right after the samples were collected and the results were recorded in the database. In this retrospective study, the results were directly acquired from the database.

### Statistical analysis

The Kolmogorov-Smirnov test was used to verify the normality of all data. Normally distributed data were expressed as means and standard deviations (SD). Abnormally distributed continuous data were expressed as medians with the 25th and 75th quartiles. Categorical data were expressed as frequency and percentage.

The risk factors of secondary infection of septic patients were explored by a two-step method. Firstly, univariate analysis was conducted. Covariates included age, gender, comorbidities, smoking history, site of primary infection, hemodynamic status and severity of disease on admission, HLA-DR expression, serum cytokines levels and clinical interventions before onset of secondary infection. Student’s t test was used to compare normally distributed data and Mann-Whitney *U* test was utilized to compare abnormally distributed data. Categorical data were compared by Pearson’s chi-square test or Fisher’s exact test when appropriate. Secondly, covariates with statistical significance in univariate analysis were tested in multivariate binary logistic regression analysis to identify the independent risk factors by means of Backward: Conditional method. Because of the data missing of HLA-DR expression and serum cytokines levels, they were not brought into multivariate analysis. Dynamic changes of HLA-DR expression and serum cytokines levels were also statistically evaluated by comparing the levels of biomarkers between different points in time using Mann-Whitney *U* test.

In our study, we treated in-hospital LOS as an outcome of secondary infection, rather than a potential risk factor. A multistate model with 4 states (state 0: admission, state 1: development of secondary infection, state 2: being discharged alive, state 3: in-hospital death) was performed using “etm” package in R in order to explore the influence of secondary infection on in-hospital LOS [3, 26], where the data of patients with an in-hospital LOS longer than 100 days were omitted to eliminate the impact of extreme cases (see Additional file 1: Figure S1). Survival analysis was conducted using Kaplan-Meier method. Log-rank test was used to compare survival curves and it was conducted in every division once two curves had intersections. The two-step method was also used to explore the risk factors of mortality. Univariate analysis was conducted first and followed by multivariate binary logistic regression analysis. Secondary infection was among covariates, together with age, gender, comorbidities, smoking history, site of primary infection, hemodynamic status and severity of disease on admission, clinical interventions and in-hospital and ICU LOS.

All statistical analyses were two-sided, and the significance level was set to *P* < 0.05. We checked the model assumptions before using each statistical method. Statistical analysis was conducted on SPSS 25.0 (SPSS Inc., IL, USA) and R 3.5.1 (R Development Core Team).

## Results

### Characteristics of septic patients

**Fig. 1:**
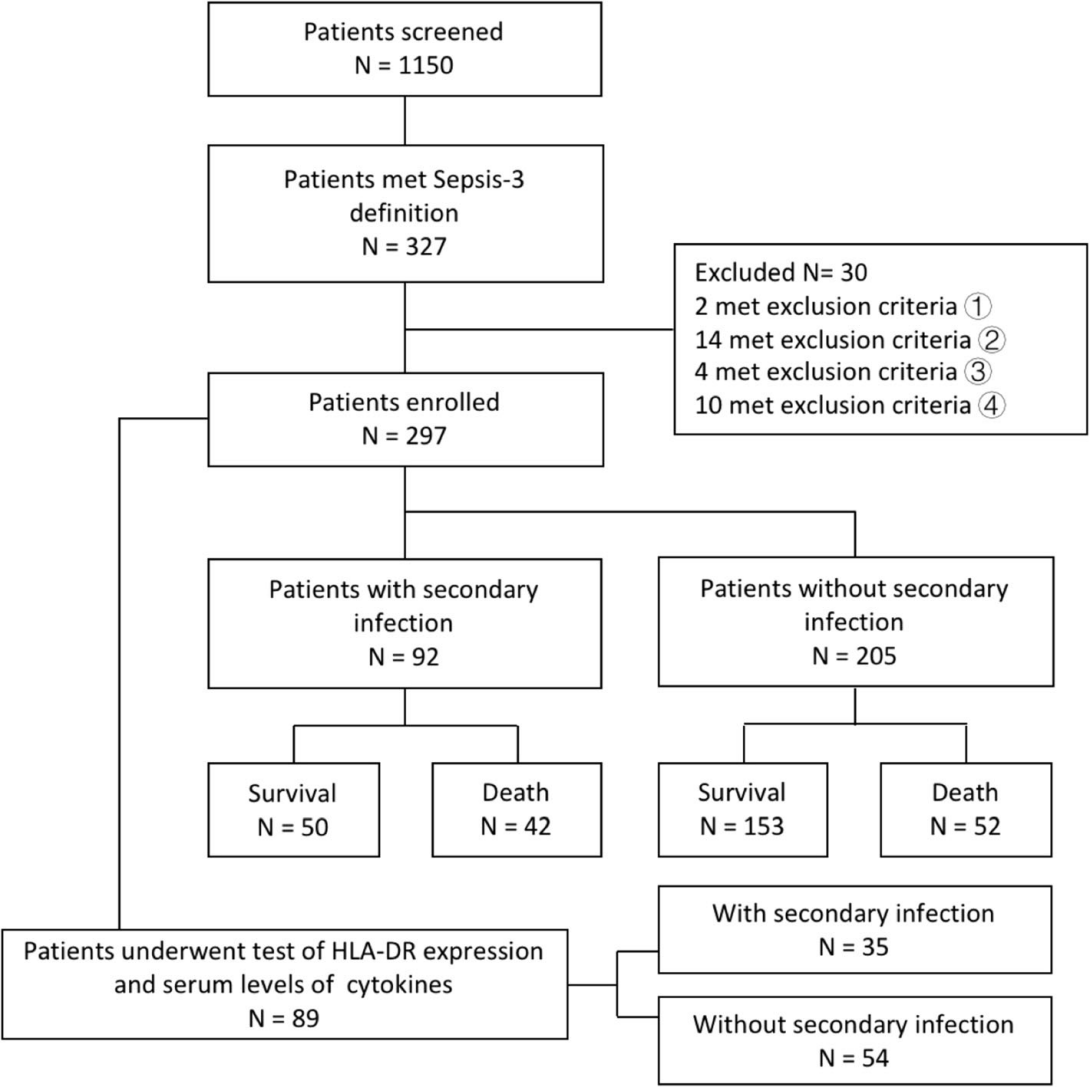
Study flowchart

From January, 2014 to January, 2018, a total of 297 patients were enrolled. A flowchart to illustrate the recruitment process was shown in Fig. 1. Among all included patients, 195 were men and the median age was 66 years. Two hundred forty-one patients had comorbidities (81.1%). Respiratory tract was the most common site of primary infection (*n* = 216, 72.7%). Other sites of infection included abdomen (*n* = 62, 20.9%), urinary tract (*n* = 22, 7.4%), skin and soft tissue (*n* = 12, 4%) and blood stream (*n* = 4, 1.3%), 21 patients had more than one infection sites (7.1%). Seventy-seven patients had septic shock on admission (25.9%). The baseline characteristics of the enrolled patients were shown in Table 1.

**Table 1:** Characteristics of septic patients classified according to developing secondary infection or not

	With secondary infection *n* = 92	Without secondary infection *n* = 205	*P* value
Baseline characteristics			
Age, median (25th,75th)	66.5 (53.5–78.8)	65 (52.3–75)	0.323
> 65 years, n (%)	50 (54.3)	105 (51.2)	0.618
Men, n (%)	63 (68.5)	132 (64.4)	0.493
Comorbidities, n (%)			
None	16 (17.4)	40 (19.5)	0.666
Hypertension	42 (45.7)	82 (40)	0.361
Other cardiovascular diseases^a^	15 (16.3)	25 (12.2)	0.337
Diabetes mellitus	23 (25)	46 (22.4)	0.629
Cerebrovascular diseases	6 (6.5)	13 (6.3)	0.953
Respiratory diseases	9 (9.8)	23 (11.2)	0.712
Hepatitis and cirrhosis	3 (3.3)	10 (4.9)	0.761
Renal insufficiency	4 (4.3)	15 (7.3)	0.334
Malignancy	8 (8.7)	17 (8.3)	0.908
Immunosuppression	12 (13)	24 (11.7)	0.744
Smoker, n (%)	32 (34.8)	69 (33.7)	0.85
Site of infection, n (%)			
Respiratory tract	70 (76.1)	146 (71.2)	0.384
Abdomen	15 (16.3)	47 (22.9)	0.194
Urinary tract	8 (8.7)	14 (6.8)	0.57
Skin and soft tissue	6 (6.5)	6 (2.9)	0.203
Blood stream	2 (2.2)	2 (1)	0.59
More than one sites	8 (8.7)	13 (6.3)	0.464
In shock on admission, n (%)	28 (30.4)	49 (22.4)	0.235
Severity of disease, median (25th,75th)			
APACHE II score	17 (9.25–22)	11 (7–18)	0.001
SOFA score	4 (3–8)	4 (2.5–6)	0.007
Monocyte HLA-DR expression (%)^b^			
Day 1, mean (SD)	31.6 (14.3)	34.5 (14.9)	0.364
Day 3, median (25th,75th)	28.6 (18.8–42)	41.1 (27.5–50.4)	0.048
Day 7, median (25th,75th)	29.6 (14.3–35.1)	33.2 (13.8–65.4)	0.722
Levels of serum cytokines (pg/ml)^b^			
Day 1, median (25th,75th)			
IL-6	26.8 (13.6–363.5)	21.1 (7.5–58.2)	0.025
IL-8	36.3 (18.7–70)	22.9 (12–77.5)	0.375
IL-10	8.6 (5.4–24)	10 (9.3–17.8)	0.121
Day 3, median (25th,75th)			
IL-6	628.5 (23.5–1694.5)	640.5 (17.8–942.3)	0.478
IL-8	29.89 (20–106)	8 (4.9–15.6)	< 0.001
IL-10	21.7 (6.4–35.3)	14.6 (5–27.7)	0.303
Day 7			
IL-6, median (25th,75th)	921 (652–1377)	754 (584–1004)	0.226
IL-8, median (25th,75th)	11.1 (6–41.8)	12.5 (5.7–14)	0.79
IL-10, mean (SD)	60.6 (47.3)	16.8 (10.4)	0.035
Interventions, n (%)^c^			
Glucocorticoid	46 (50)	80 (39)	0.077
Anticoagulation therapy	33 (35.9)	66 (32.2)	0.535
Mechanical ventilation	68 (74)	104 (50.7)	< 0.001
Urinary catheterization	72 (78.3)	83 (40.5)	< 0.001
Deep venous catheterization	66 (71.7)	74 (36.1)	< 0.001
Continuous renal replacement therapy	11 (12)	19 (9.3)	0.477
Blood transfusion	25 (27.2)	30 (14.6)	0.011
LOS (days), median (25th,75th)			
In-hospital	23.5 (12–34)	22 (10–32.5)	< 0.001
ICU	11 (7–17)	11 (6–16.5)	< 0.001
Mortality, n (%)			
In-hospital	42 (45.7)	52 (25.4)	0.001
30-day	32 (34.8)	48 (23.4)	0.041
90-day	39 (42.4)	52 (25.4)	0.003

^a^Other cardiovascular diseases included coronary heart disease, arrhythmia, myocardiosis and valvular heart disease

^b^Data of 89, 77 and 21 patients were available for HLA-DR expression at day 1, 3 and 7 respectively, in which 35, 34 and 12 patients developed secondary infection. And data of 87, 38 and 18 patients were available for cytokines at day 1, 3 and 7 respectively, in which 33, 18 and 8 patients developed secondary infection

^c^In the group of secondary infection, it referred to the interventions before the onset of secondary infection

### Characteristics of septic patients with secondary infection

One hundred fifty cases of secondary infection were developed in 92 patients, 26 of whom had multiple secondary infections. Respiratory tract was the most common site of secondary infection (*n* = 84, 56%), followed by urinary tract (*n* = 42, 28%), blood stream and disseminated infection (*n* = 18, 12%), abdomen (*n* = 5, 3.3%) and skin and soft tissue (*n* = 1, 0.7%). Day 8 was the median time of developing the first secondary infection. *Acinetobacter baumanii* (*n* = 40, 26.7%), *Klebsiella pneumoniae* (*n* = 21, 14%), *Enterococcus faecium* (*n* = 11, 7.3%), *Candida tropicalis* (*n* = 9, 6%), *Pseudomonas aeruginosa* (*n* = 9, 6%) and *Staphylococcus aureus* (*n* = 9, 6%) were common identified pathogens. In 23 cases, pathogens were not identified. The characteristics of secondary infections were shown in Table 2 and time of onset, distribution of pathogen and diagnostic criterion of each infection were shown in Additional file 2: Table S1.

**Table 2:** Characteristics of secondary infections

Site of infection, n (%) ^a^	
Respiratory tract	
PNU	83 (55.3)
LUNG	1 (0.7)
Urinary tract	
SUTI	41 (27.3)
OUTI	1 (0.7)
Blood stream and disseminated infection	
LCBI	12 (8)
DI	6 (4)
Abdomen	
IAB	4 (2.7)
GIT	1 (0.7)
Skin and soft tissue	
ST	1 (0.7)
Time of onset of the first identified secondary infection	
Median (25th,75th)	8 (5.25,14)
Time range, n (%)	
day 3	5 (5.4)
> day 3, ≤day 7	36 (39)
> day 7, ≤day 15	33 (35.9)
> day 15	18 (19.6)
Patients with multiple secondary infections, n (%)	26 (28.3)
Secondary infection without identified pathogens, n (%)	23 (15.3)

^a^Diagnosis was according to CDC/NHSN criteria [25]. PNU Pneumonia, LUNG Other infections of the lower respiratory tract, SUTI Symptomatic urinary tract infection, OUTI Other infections of the urinary tract, DI Disseminated infection, GIT Gastrointestinal tract, IAB Intraabdominal infection, LCBI Laboratory-confirmed bloodstream infection, ST Soft tissue infection

### Risk factors of secondary infection in septic patients

No statistical significance existed between septic patients with and without secondary infection concerning age, gender, comorbidity and site of primary infection. In univariate analysis, statistical significance was found in severity of illness on admission (APACHE II score: *P* = 0.001; SOFA score: *P* = 0.007) and some interventions before the onset of secondary infection such as the use of mechanical ventilation (OR 2.752, 95% CI 1.604 to 4.721, *P* < 0.001), urinary catheterization (OR 5.292, 95% CI 2.997 to 9.343, *P* < 0.001), deep venous catheterization (OR 4.494, 95% CI 2.629 to 7.680, *P* < 0.001) and blood transfusion (OR 2.152, 95% CI 1.18 to 3.925, *P* = 0.011) (Table 1). Factors with statistical significance were tested under multivariate logistic regression analysis and urinary catheterization (OR 3.384, 95% CI 1.791 to 6.392, *P* < 0.001) and deep venous catheterization (OR 2.608, 95% CI 1.422 to 4.784, *P* = 0.002) remained statistical significant (Table 3).

**Table 3:** Results of multivariate logistic regression test of the risk factors of secondary infection

Variables^a^	Partial regression coefficient	Standard error	Wald χ2	*P* value	OR	95% CI
Urinary catheterization	1.219	0.325	14.109	< 0.001	3.384	1.791–6.392
Deep venous catheterization	0.959	0.309	9.601	0.002	2.608	1.422–4.784

^a^Analysis was conducted by method Backward: Conditional. Variable blood transfusion was removed on step 2, mechanical ventilation on step 3, APACHE II score on step 4 and SOFA score on step 5

### The association between immune dysfunction and secondary infection of sepsis

**Fig. 2:**
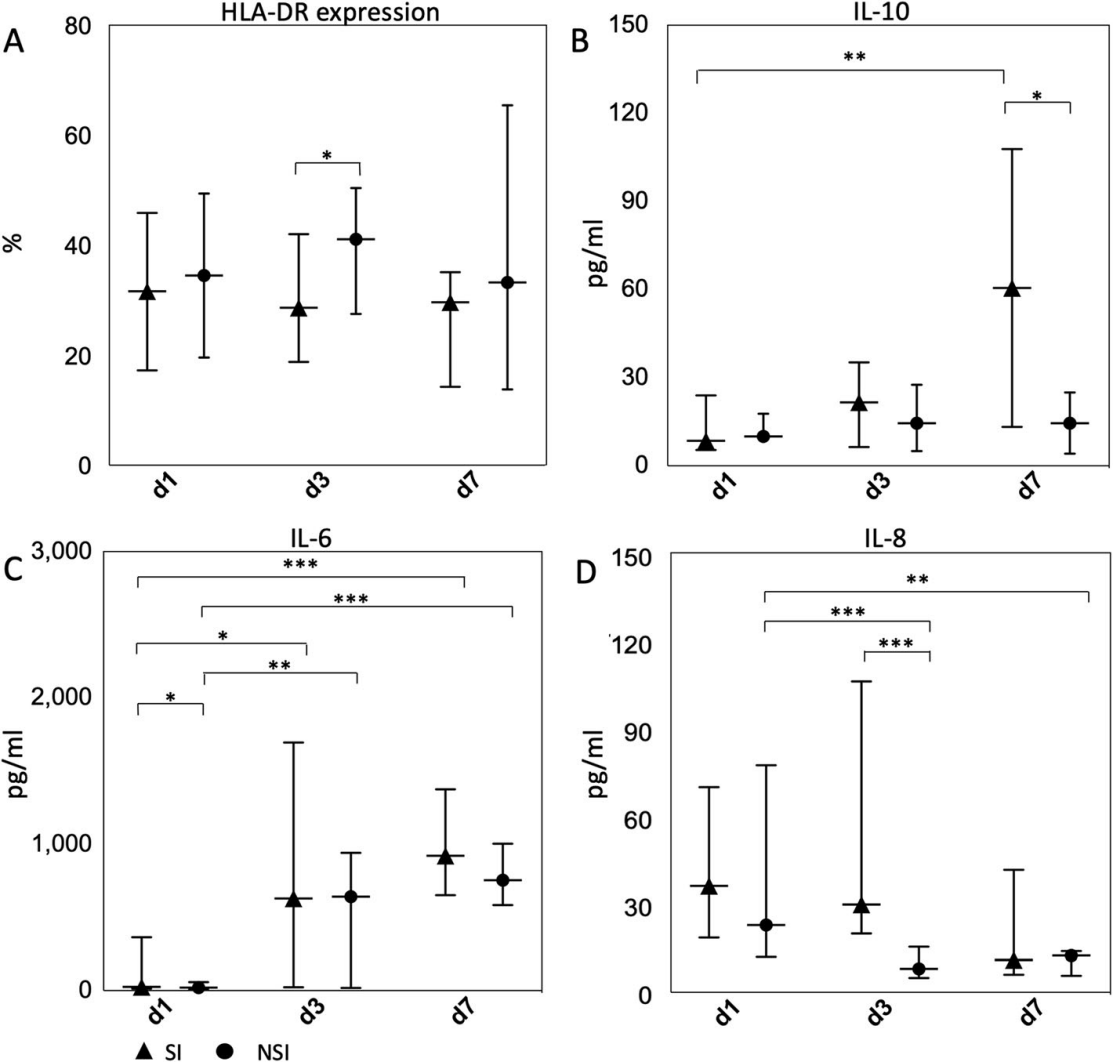
Biomarkers of immune status in septic patients stratified according to developing secondary infection or not. Data of a part of patients were available for HLA-DR expression and cytokines and the exact numbers were shown in Table 1. Data were presented as medians (shown as triangles or circles) and 25- and 75- percentile error bars. Exceptions were mean and standard deviation error bars were used in HLA-DR expression at day 1 and IL-10 level at day 7. **a** and **b** represented the levels and dynamic changes of two anti-inflammatory biomarkers (HLA-DR and IL-10) respectively. **c** and **d** represented the levels and dynamic changes of two pro-inflammatory biomarkers (IL-6 and IL-8) respectively. * *P* < 0.05, ** *P* < 0.01, *** *P* < 0.001. SI, secondary infection; NSI, non-secondary infection

**Fig. 3:**
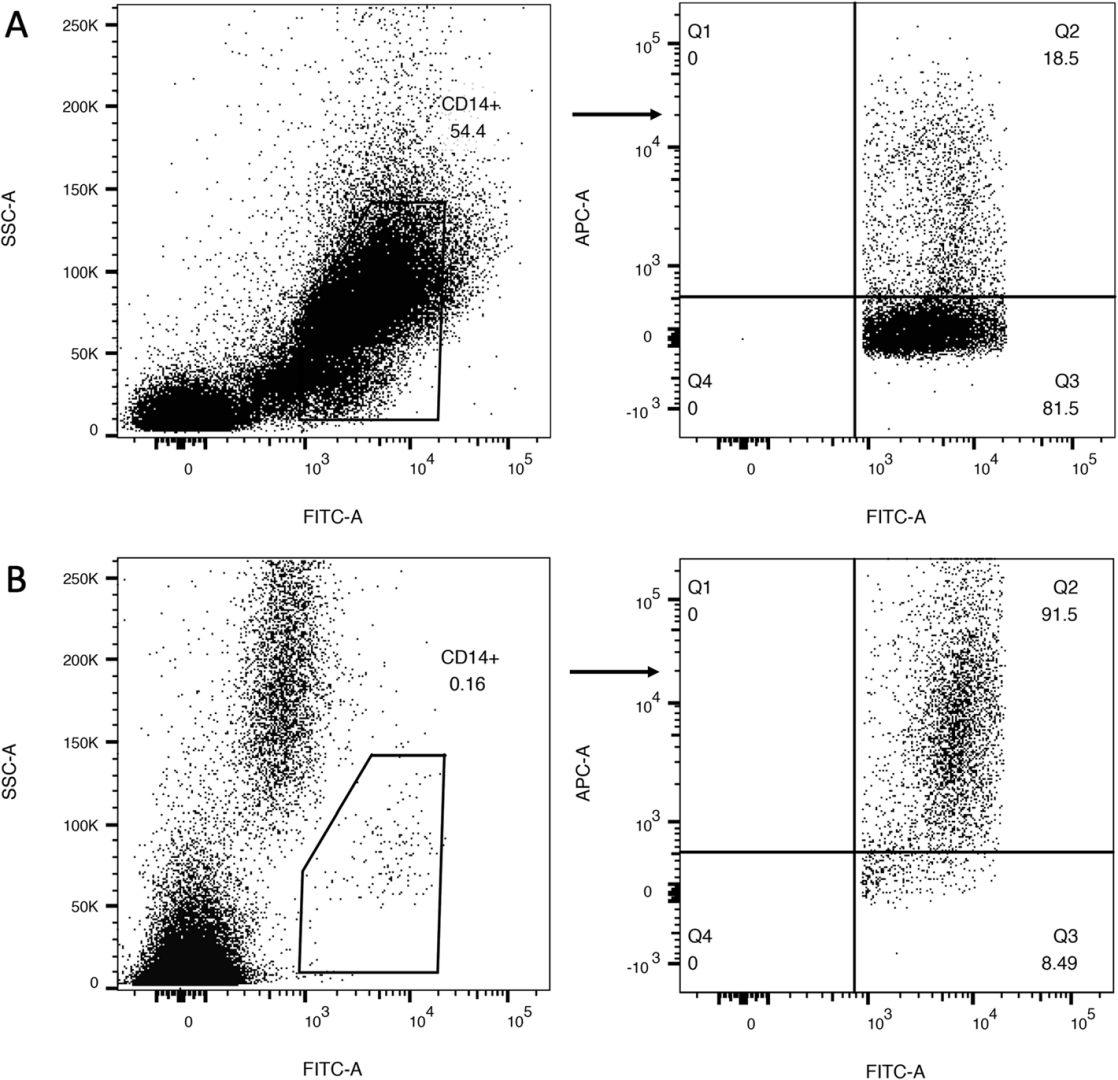
Representative plots of monocyte HLA-DR measurement by flow cytometry. Monocyte HLA-DR expression was measured by flow cytometry. The samples were collected at day 3 after admission. **a** The left dot-plot (SSC vs. FITC) delimited the monocytic region. The right dot-plot (APC vs. FITC) delimited the CD14^+^ HLA-DR^+^ monocyte (upper right region). The analysis was performed on a patient with immunosuppression as was reflected by the decreased proportion of CD14^+^ HLA-DR^+^ monocyte (18.5%). **b** The same strategy of analysis was used on a patient without immunosuppression. FITC, fluorescein isothiocyanate; APC, allophycocyanin; SSC, side scatter

Data of a part of patients were available for HLA-DR expression and cytokines. The exact numbers were shown in the legend of Table 1. In the univariate analysis of the risk factors of secondary infection, statistical significance was found in HLA-DR expression at day 3 (*P* = 0.048), IL-6 level at day 1 (*P* = 0.025), IL-8 level at day 3 (*P* < 0.001) and IL-10 level at day 7 (*P* = 0.035). The results were shown in Table 1 and Fig. 2. Although statistical significance was not found at every time point, a trend of decrease of HLA-DR expression and increase of IL-10 level in secondary infection group was observed, which is indicative of immunosuppression (Fig. 2a and b). Interestingly, a reverse trend of dynamic change was found between two pro-inflammatory cytokines IL-6 and IL-8 in both secondary infection and non-secondary infection groups (Fig. 2c and d). Dynamic changes of those markers were statistically significant between certain points in time, the results were shown in Fig. 2 and Additional file 3: Table S2. Representative flow cytometry profiles for HLA-DR expression were shown in Fig. 3.

### The association between secondary infection and the outcomes of sepsis

**Fig. 4:**
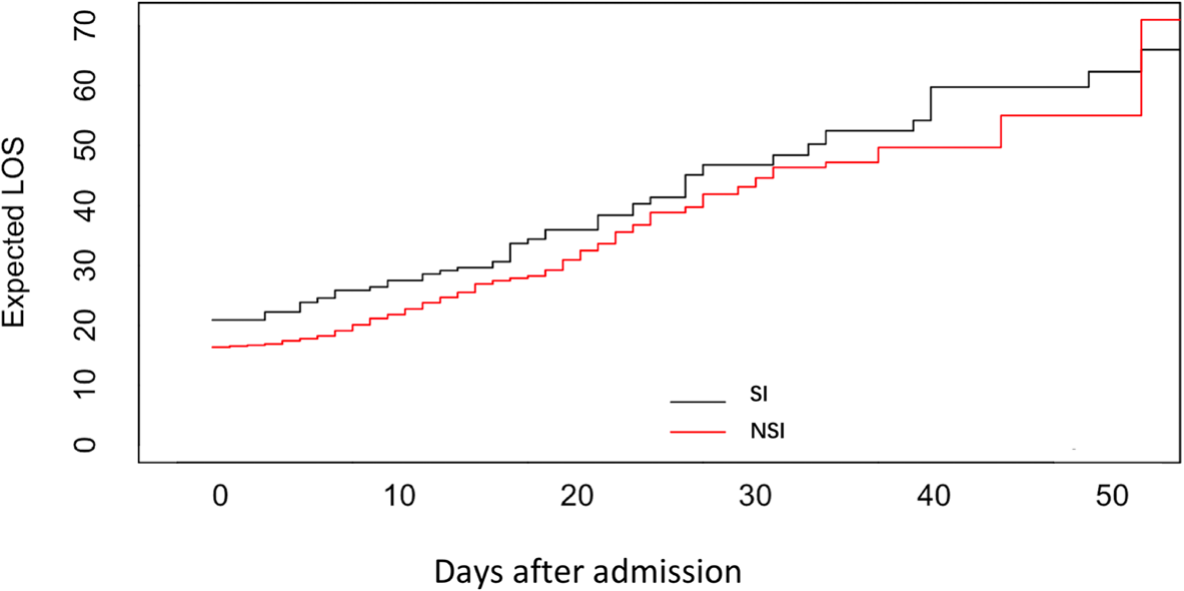
Expected length of stay of septic patients with and without secondary infection. SI, secondary infection; NSI, non-secondary infection

**Fig. 5:**
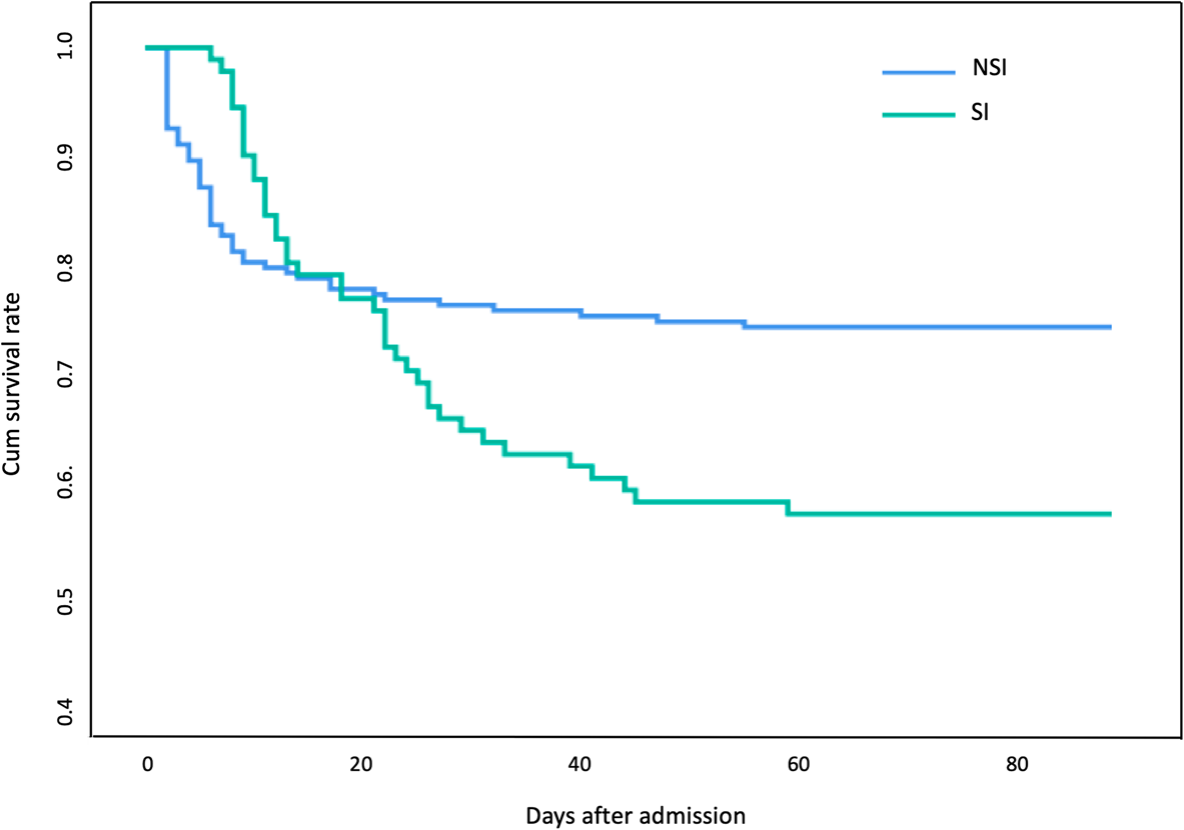
Kaplan-Meier survival curves of overall septic patients before day 90. SI, secondary infection; NSI, non-secondary infection

Secondary infection group had longer LOS in hospital and ICU than non-secondary infection group (in-hospital LOS: *P* < 0.001; ICU LOS: *P* < 0.001) **(**Table 1**)**. Multistate model revealed expected prolonged LOS in hospital was 4.63 days based on a standard error of 1.87 days **(**Fig. 4**)**. In-hospital, 30-day, 90-day mortality was 45.7, 34.8, 42.4% in secondary infection group and 25.4, 23.4 and 25.4% in non-secondary infection group respectively (OR 2.472, 95% CI 1.474 to 4.145, *P* = 0.001; OR 1.744, 95% CI 1.019 to 2.985, *P* = 0.041; OR 2.165, 95% CI 1.288 to 3.640, *P* = 0.003, respectively). The proportion of developing secondary infection were 44.7 and 24.6% in in-hospital mortality group and survival group respectively (OR 2.472, 95% CI 1.474 to 4.145, *P* = 0.001) (see Additional file 4: Table S3). Multivariate binary logistic regression analysis also found out that secondary infection was an independent risk factor of in-hospital mortality (OR 3.476, 95% CI 1.599 to 8.219, *P* = 0.003) (see Additional file 5: Table S4). Kaplan-Meier survival curves and Log-rank test revealed no difference between two groups before day 15 (*P* = 0.426) (see Additional file 6: Figure S2). But non-secondary infection group had a better survival between day 15 and day 90 (*P* < 0.001) **(**Fig. 5**)** and subgroup analysis showed that the difference remained significant in both groups of patients with and without septic shock (*P* = 0.04 and *P* < 0.001) (see Additional file 7: Figure S3).

## Discussion

Our study confirmed a high incidence of secondary infection in septic patients (31.0%) and suggested urinary and deep venous catheterization could bring higher risk of developing secondary infection, in which immunosuppression might be the underlying mechanism. Secondary infection also affected the outcomes, which featured poor survival at later period (> 15 days after admission) and expected prolonged in-hospital LOS of 4.63 ± 1.87 days.

We found that secondary infections mostly developed in respiratory tract and were caused by Gram-negative bacteria. This finding was consistent with previous studies. A recent meta-analysis revealed that lower respiratory tract was the most common site of nosocomial infection in general hospital and *Pseudomonas aeruginosa, Escherichia coli, Acinetobacter baumannii* and *Klebsiella pneumoniae* were among most common pathogens [27]. There’s a study suggesting that the high pathogenicity of such Gram-negative bacteria was due to drug resistant and invasive procedures which served as approaches of the invasion of pathogens [28]. It has been increasingly acknowledged that increased susceptibility of secondary infection could be pathogen-specific due to the different patterns of immune barrier destruction which caused opportunistic bacterial and fungal infections, as well as higher chance of viral reactivation and co-infection [29–31]. In our study, 6 cases of secondary infections were defined as disseminated infections, which were likely caused by viruses according to the CDC criteria [25]. However, it’s possible that some pathogenic microorganisms, especially viruses, were not identified due to limited testing technologies.

We found higher APACHE II and SOFA scores on admission in patients with secondary infection, which were similar to previous studies [3, 4, 32]. Although illness severity was not found to be an independent risk factor of secondary infection in this study, it could be explained that the more severe patients died mostly at the very early period of disease before developing secondary infections, which might impact the true association between the risk and illness severity.

It’s widely acknowledged that catheter indwelling was a major cause of nosocomial infection [33–35]. We found that urinary catheterization was an independent risk factor of secondary infection. Another study revealed that catheter-associated urinary tract infection was not only affected by duration of urinary catheterization, but also the presence of another site of nosocomial infection [36], which was confirmed by our study that many cases of secondary infections in urinary tract were subsequent to secondary infections at other sites. Deep venous catheterization was also common in ICU setting and our finding was consistent with the study by van Vught et al. that it was also an independent risk factor of secondary infection [4]. It’s also proved by previous studies that the need for mechanical ventilation of critical ill patients incurred high prevalence of ventilator-associated pneumonia, which accounted for nearly half of nosocomial infections [3, 4, 37]. Blood transfusion was also a potential risk factor due to the effect of transfusion-related immune modulation (TRIM) as reveal by previous studies [38–41]. Nevertheless, mechanical ventilation and blood transfusion were only found to be risk factors in univariate analysis of our study, but not multivariate analysis. It might be explained by the lack of the discrimination of invasive and non-invasive ventilation, the length of ventilation and the quantity and type of blood transfusion due to limited medical records.

Immune status of septic patients and its underlying mechanism have been widely studied. Innate immune function was compromised due to the dysfunction of neutrophils, monocytes, dendritic cells and myeloid-derived suppressor cells (MDSCs) which caused altered first-line of defense, inhibition of T cell proliferation, altered inflammatory response and incomplete activation of T cells [8]. Adaptive immune function was also compromised as sepsis affected the effector functions and phenotypes of T cells, B cells and innate-type lymphocytes [8]. HLA-DR and cytokines were chosen to reflect the immune status in this study. HLA-DR was a marker reflecting both innate and adaptive immune function and lower expression indicated immunosuppression [8]. IL-10 was an anti-inflammatory cytokine and elevated level reflected the down-regulation of inflammation process. It might generate MDSCs and enhance the immunosuppression during sepsis [20, 42]. In secondary infection group of this study, HLA-DR expression was lower and IL-10 level showed a trend of increase, which was a sign of immunosuppression. A more severe pro-inflammatory response in secondary infection group presented as higher levels of IL-6 and IL-8, was also observed in this study. This confirmed the previous conception that higher pro- and anti- inflammatory processes might exist at the same time in septic patients with secondary infection [21, 23]. Interestingly, we observed a reverse trend of dynamic change between IL-6 and IL-8, though they were both pro-inflammatory cytokines. This might be explained by that the increase of IL-6 demonstrated the progress of inflammation, as the blood sample collected at day 3 and 7 were more often from severely ill patients. IL-8, as we hypothesized, might be involved in early phase inflammatory process rather than later phase and thus showed a trend of decrease. As the dynamic changes were only statistical significant between certain points in time, studies with larger sample size are necessary to further the study. Those results enlightened us that the identification and risk stratification of immunosuppression and the therapies that boost immunity could be beneficial to the prevention of secondary infection [13, 43, 44].

We found that secondary infection prolonged the hospitalization time using a multistate model, which could be a result of the complexity of disease requiring longer in-hospital treatment and longer LOS in turn increased the risk of secondary infection. Multivariate analysis of our study also revealed that secondary infection was an independent risk factor of in-hospital death. Survival analysis further demonstrated that patients with secondary infection had worse prognosis after first 15 days. In the first 15 days, secondary infection group even had better survival and this could be explained by that patients who were severely sick died earlier before they developed secondary infections. This was consistent with the previous concept that the mortality of patients who survived that early period was more likely affected by secondary infection [13]. A re-increased microbiological burden revealed by positive blood cultures at later phase of sepsis (> 15 days) was observed in the study by Otto et al., which was indicative of secondary infection and poor outcomes [45]. However, Goldenberg et al. addressed that secondary infection was not the main cause of death in sepsis as they found only a small portion (14%) of septic patients died with an evidence of secondary infection. Some studies found that mitochondrial dysfunction, microvascular leak or even activity of daily living could serve as causes of death from sepsis [5, 28].

This study had some limitations. First, the sample size was relatively small as a single-center study. Second, some clinical data such as the use of antibiotics, the exact dose of glucocorticoids, duration of mechanical ventilation and catheter indwelling were not documented due to the limited medical records, which blocked us from exploring the dose-response relationship. Third, data of HLA-DR expression and serum cytokines levels of many patients were not available as a retrospective study. Thus, the clinical characteristics, risk factors, immune status and prognosis of secondary infection of sepsis were worthy of further prospective research with a larger sample size.

## Conclusions

Invasive operations such as urinary catheterization and deep venous catheterization increased the risk of developing secondary infection, in which underlying immunosuppression also played a role. Secondary infection affected outcomes of patients as it prolonged expected in-hospital LOS and increased mortality in patients who survived early period of sepsis. The monitoring of immune status and proper care to minimize the invasion of pathogens were keys to lower incidence of secondary infection.

## Supplementary information

**Additional file 1:** **Figure S1.** Illustration of multistate model to explore the expected length of stay. Patients without secondary infection would move from state 0 to state 2 or state 3. Patients with secondary infection would move from state 0 to state 1, and then to state 2 or state 3.

**Additional file 2:** **Table S1.** Time of onset, pathogen and diagnostic criterion of each secondary infection.

**Additional file 3:** **Table S2.** Results of the comparison of the change of HLA-DR expression and serum cytokines levels.

**Additional file 4:** **Table S3.** Characteristics of the septic patients classified according to the prognosis.

**Additional file 5:** **Table S4.** Results of multivariate logistic regression test of risk factors of the in-hospital death.

**Additional file 6:** **Figure S2.** Kaplan-Meier survival curves of septic patients after admission. (A) Survival curves of overall septic patients before day 15; (B) Survival curves of septic patients without septic shock before day 15; (C) Survival curves of septic patients with septic shock before day 15; (D) Survival curves of overall septic patients before day 30; (E) Survival curves of septic patients without septic shock before day 30; (F) Survival curves of septic patients with septic shock before day 30; (G) Survival curves of septic patients without septic shock before day 90; (H) Survival curves of septic patients with septic shock before day 90.

**Additional file 7:** **Figure S3.** Kaplan-Meier survival curves of septic patients after day 15. Cumulative survival rate was considered as 1 at day 15. (A) Survival curves of overall septic patients between day 15 and 30; (B) Survival curves of septic patients without septic shock between day 15 and 30; (C) Survival curves of septic patients with septic shock between day 15 and 30; (D) Survival curves of overall septic patients between day 15 and 90; (E) Survival curves of septic patients without septic shock between day 15 and 90; (F) Survival curves of septic patients with septic shock between day 15 and 90.

## Data Availability

The datasets used and/or analyzed during the current study are available from the corresponding author on reasonable request.

## Abbreviations

APACHE II: Acute Physiology and Chronic Health Evaluation II
APC: Allophycocyanin
CDC: Centers for Disease Control and Prevention
CI: Confidential interval
CKD: Chronic kidney disease
CPOE: Computerized Physician Order Entry
ELISA: Enzyme-linked immunosorbent assay
EMRS: Electronic Medical Record System
FITC: Fluorescein-5-isothiocyanate
HLA-DR: Human leukocyte antigen-D related
ICU: Intensive care unit
IL: Interleukin
LOS: Length of stay
MDSC: Myeloid-derived suppressor cells
MODS: Multiple organ dysfunction syndrome
NHSH: National Healthcare Safety Network
SD: Standard deviation
SOFA: Sequential Organ Failure Assessment
TNF-α: Tumor necrosis factor-α
TRIM: Transfusion-related immune modulation
