# Supplementary figures and images for: Clinical characteristics, risk factors, immune status and prognosis of secondary infection of sepsis: a retrospective observational study
Source: BMC Anesthesiol. 2019 Oct 18;19:185. doi: 10.1186/s12871-019-0849-9 (PMC6800505; doi:10.1186/s12871-019-0849-9)

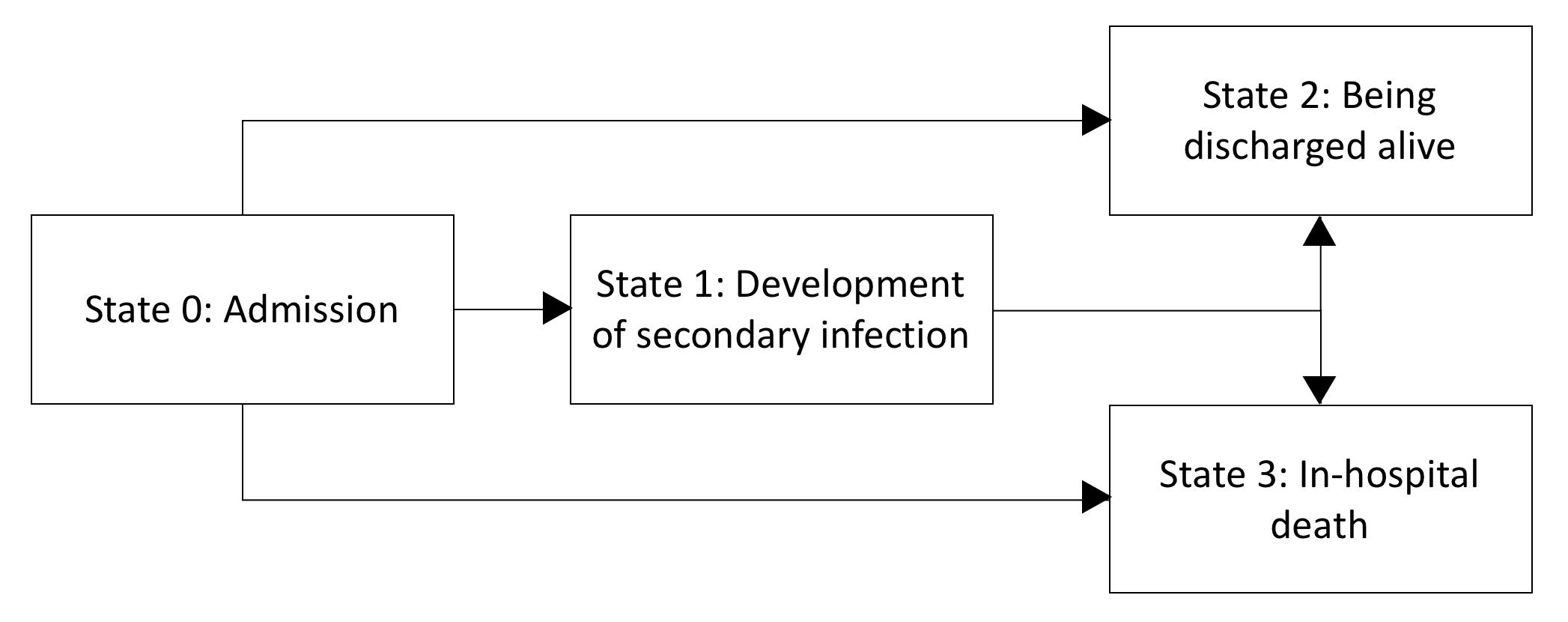

Supplement: Supplementary file 1 — Additional file 1: Figure S1. Illustration of multistate model to explore the expected length of stay. Patients without secondary infection would move from state 0 to state 2 or state 3. Patients with secondary infection would move from state 0 to state 1, and then to state 2 or state 3. [file 12871_2019_849_MOESM1_ESM.jpg]

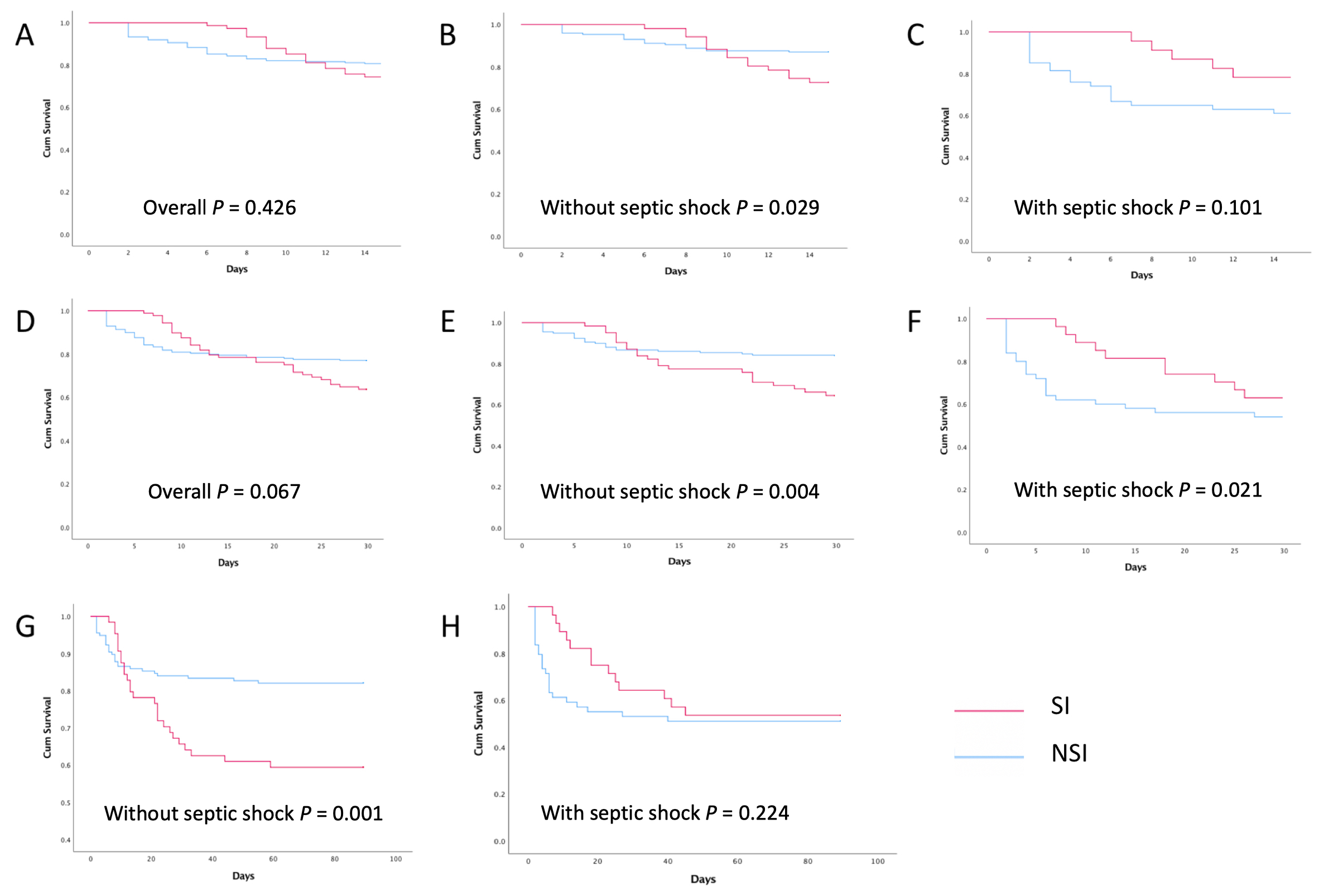

Supplement: Supplementary file 6 — Additional file 6: Figure S2. Kaplan-Meier survival curves of septic patients after admission. (A) Survival curves of overall septic patients before day 15; (B) Survival curves of septic patients without septic shock before day 15; (C) Survival curves of septic patients with septic shock before day 15; (D) Survival curves of overall septic patients before day 30; (E) Survival curves of septic patients without septic shock before day 30; (F) Survival curves of septic patients with septic shock before day 30; (G) Survival curves of septic patients without septic shock before day 90; (H) Survival curves of septic patients with septic shock before day 90. [file 12871_2019_849_MOESM6_ESM.jpg]

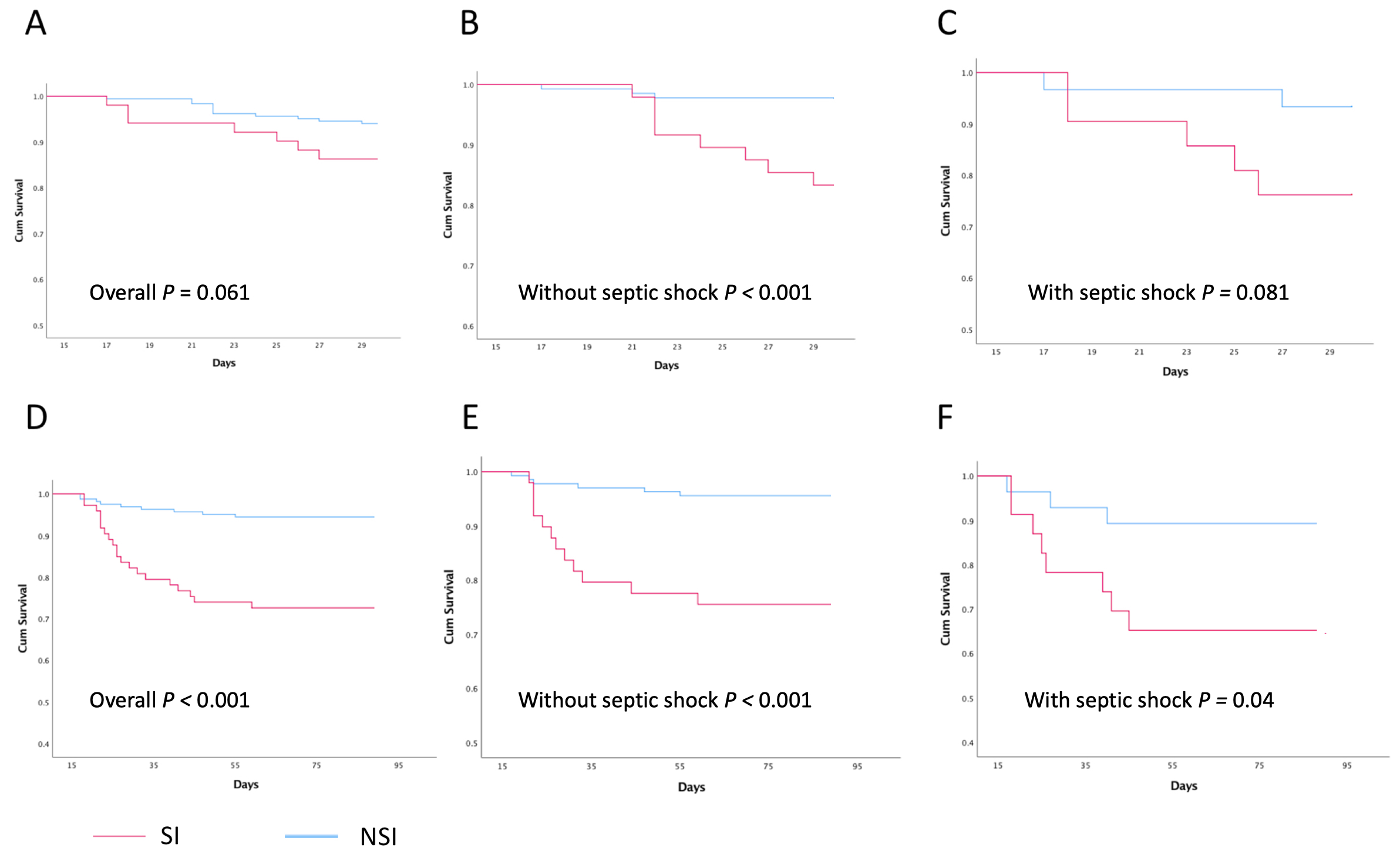

Supplement: Supplementary file 7 — Additional file 7: Figure S3. Kaplan-Meier survival curves of septic patients after day 15. Cumulative survival rate was considered as 1 at day 15. (A) Survival curves of overall septic patients between day 15 and 30; (B) Survival curves of septic patients without septic shock between day 15 and 30; (C) Survival curves of septic patients with septic shock between day 15 and 30; (D) Survival curves of overall septic patients between day 15 and 90; (E) Survival curves of septic patients without septic shock between day 15 and 90; (F) Survival curves of septic patients with septic shock between day 15 and 90. [file 12871_2019_849_MOESM7_ESM.jpg]
