# Supplementary material for: Clinical characteristics, risk factors, immune status and prognosis of secondary infection of sepsis: a retrospective observational study
Source: BMC Anesthesiol. 2019 Oct 18;19:185. doi: 10.1186/s12871-019-0849-9 (PMC6800505; doi:10.1186/s12871-019-0849-9)
Supplement: Supplementary file 2 — Additional file 2: Table S1. Time of onset, pathogen and diagnostic criterion of each secondary infection. [file 12871_2019_849_MOESM2_ESM.docx]

| **Table S1. Time of onset, pathogen and diagnostic criterion of each secondary infection** | | | |
| --- | --- | --- | --- |
| Patient No. | Time of onset (Days) | Pathogen | Diagnostic criterion ^a^ |
| 1 | 3 | *Acinetobacter Baumanii* | PNU-3 |
|  | 13 | *Stenotrophomonas maltophilia* | PNU-3 |
|  | 18 | *Klebsiella pneumoniae* | PNU-3 |
| 2 | 3 | *Staphylococcus aureus* | PNU-2 |
| 3 | 3 | *Acinetobacter Baumanii* | PNU-2 |
|  | 20 | *Pseudomonas aeruginosa* | PNU-2 |
|  | 22 | *Pseudomonas aeruginosa* | PNU-2 |
|  | 25 | *Klebsiella pneumoniae* | PNU-2 |
| 4 | 3 | *Acinetobacter Baumanii* | PNU-2 |
| 5 | 3 | not identified | DI |
| 6 | 4 | *Acinetobacter Baumanii* | PNU-2 |
|  | 5 | *Klebsiella pneumoniae* | PNU-2 |
|  | 92 | *Proteus mirabilis* | SUTI-1 |
|  | 117 | *Proteus mirabilis* | PNU-2 |
| 7 | 4 | *Acinetobacter Baumanii* | PNU-2 |
|  | 28 | *Candida tropicalis* | SUTI-1,2 |
| 8 | 4 | *Staphylococcus aureus* | PNU-2 |
|  | 24 | *Klebsiella pneumoniae* | PNU-2 |
|  | 37 | *Acinetobacter Baumanii* | PNU-2 |
| 9 | 4 | *Candida albicans* | SUTI-1 |
|  | 13 | *Acinetobacter Baumanii* | PNU-2 |
| 10 | 4 | *Klebsiella pneumoniae* | PNU-2 |
|  | 18 | *Acinetobacter Baumanii* | PNU-2 |
|  | 20 | *Escherichia coli* | SUTI-1 |
|  | 22 | *Klebsiella pneumoniae* | LCBI -1,2 |
| 11 | 4 | *Staphylococcus aureus* | PNU-2 |
| 12 | 4 | not identified | PNU-1 |
| 13 | 4 | not identified | DI |
| 14 | 4 | *Acinetobacter Baumanii* | PNU-3 |
| 15 | 4 | *Klebsiella pneumoniae/ Staphylococcus aureus* | PNU-2 |
|  | 15 | *Enterococcus faecium* | SUTI-1,2 |
| 16 | 4 | not identified | PNU-1 |
| 17 | 5 | *Candida albicans* | SUTI-1,2 |
|  | 8 | *Candida albicans* | OUTI-1,2 |
| 18 | 5 | *Candida glabrata* | SUTI-1 |
|  | 7 | *Stenotrophomonas maltophilia* | PNU-2 |
|  | 16 | *Burkholderia cepacia* | PNU-2 |
|  | 17 | *Pseudomonas aeruginosa* | PNU-2 |
|  | 19 | *Acinetobacter Baumanii* | PNU-2 |
| 19 | 5 | not identified | PNU-1 |
| 20 | 5 | *Enterococcus faecium* | SUTI-1 |
| 21 | 5 | not identified | PNU-1 |
| 22 | 5 | not identified | PNU-1 |
| 23 | 5 | *Pseudomonas aeruginosa* | PNU-2 |
| 24 | 6 | *Pseudomonas aeruginosa* | PNU-2 |
|  | 11 | *Serratia marcescens* | PNU-2 |
| 25 | 6 | not identified | PNU-1 |
| 26 | 6 | *Acinetobacter Baumanii* | PNU-2 |
| 27 | 6 | *Serratia marcescens* | PNU-2 |
| 28 | 6 | *Acinetobacter Baumanii* | LUNG-1 |
| 29 | 6 | *Enterococcus faecium* | SUTI-1,2 |
| 30 | 6 | *Enterococcus faecium* | SUTI-1,2 |
| 31 | 6 | not identified | DI |
| 32 | 6 | *Enterococcus faecium* | SUTI-1,2 |
| 33 | 7 | *Klebsiella pneumoniae* | PNU-2 |
|  | 10 | *Klebsiella pneumoniae* | SUTI-1,2 |
|  | 14 | *Klebsiella pneumoniae* | LCBI -1,2 |
|  | 25 | *Acinetobacter Baumanii* | PNU-2 |
| 34 | 7 | *Stenotrophomonas maltophilia* | PNU-2 |
| 35 | 7 | *Candida tropicalis* | SUTI-1 |
| 36 | 7 | *Acinetobacter Baumanii* | PNU-2 |
| 37 | 7 | *Klebsiella pneumoniae* | PNU-2 |
| 38 | 7 | *Acinetobacter Baumanii* | LCBI -1,2 |
| 39 | 7 | *Klebsiella pneumoniae* | PNU-2 |
| 40 | 7 | *Acinetobacter Baumanii* | PNU-2 |
| 41 | 7 | not identified | PNU-1 |
| 42 | 8 | *Acinetobacter Baumanii* | LCBI -1 |
|  | 9 | *Candida tropicalis* | SUTI-1,2 |
| 43 | 8 | not identified | PNU-1 |
| 44 | 8 | *Acinetobacter Baumanii* | SUTI-1,2 |
| 45 | 8 | not identified | GIT-1,2 |
| 46 | 8 | not identified | DI |
| 47 | 8 | *Acinetobacter Baumanii/ Aspergillus fumigatus* | PNU-2 |
| 48 | 8 | *Staphylococcus aureus* | PNU-2 |
| 49 | 9 | *Klebsiella pneumoniae* | LCBI -1,2/IAB-3 |
| 50 | 9 | not identified | DI |
| 51 | 9 | not identified | PNU-1 |
| 52 | 9 | *Candida tropicalis* | SUTI-1 |
| 53 | 10 | *Acinetobacter Baumanii* | PNU-2 |
| 54 | 10 | not identified | PNU-1 |
| 55 | 11 | *Acinetobacter Baumanii* | PNU-2 |
|  | 20 | *Klebsiella pneumoniae* | PNU-2 |
| 56 | 11 | *Candida albicans* | SUTI-1 |
| 57 | 11 | *Candida albicans* | SUTI-1,2 |
| 58 | 12 | not identified | IAB-3 |
|  | 9 | *Staphylococcus aureus* | PNU-2 |
|  | 12 | *Enterococcus faecium* | SUTI-1,2 |
| 59 | 12 | *Candida glabrata* | SUTI-1,2 |
|  | 16 | *Candida tropicalis* | ST-1,2,4 |
|  | 16 | *Candida tropicalis/Acinetobacter Baumanii* | PNU-2 |
|  | 24 | *Pseudomonas aeruginosa* | SUTI-1 |
|  | 44 | *Pseudomonas aeruginosa/Klebsiella pneumoniae* | PNU-2 |
|  | 72 | *Enterococcus faecium/Staphylococcus haemolyticus* | LCBI -1,2 |
|  | 73 | *Candida tropicalis* | SUTI-1,2 |
| 60 | 12 | *Pseudomonas aeruginosa* | PNU-2 |
|  | 13 | *Candida parapsilosis* | SUTI-1 |
| 61 | 12 | *Stenotrophomonas maltophilia* | PNU-2 |
| 62 | 13 | *Klebsiella pneumoniae* | PNU-2 |
| 63 | 13 | *Klebsiella pneumoniae* | SUTI-1,2 |
| 64 | 13 | *Acinetobacter Baumanii* | PNU-2 |
| 65 | 13 | not identified | PNU-1 |
| 66 | 14 | *Candida albicans* | LCBI -1,2 |
|  | 44 | *Staphylococcus aureus* | PNU-2 |
|  | 47 | *Pseudomonas aeruginosa* | PNU-2 |
|  | 51 | *Acinetobacter Baumanii* | PNU-2 |
| 67 | 14 | *Acinetobacter Baumanii* | PNU-2/SUTI-1 |
| 68 | 14 | not identified | DI |
| 69 | 14 | *Acinetobacter Baumanii* | PNU-2 |
| 70 | 14 | not identified | SUTI-2 |
| 71 | 15 | *Enterococcus faecium* | SUTI-1,2 |
|  | 19 | *Candida albicans* | SUTI-1,2 |
|  | 33 | *Escherichia coli* | SUTI-1,2 |
| 72 | 15 | *Acinetobacter Baumanii* | PNU-2 |
| 73 | 15 | *Staphylococcus aureus* | PNU-2 |
| 74 | 15 | *Acinetobacter Baumanii* | PNU-2 |
| 75 | 16 | *Acinetobacter Baumanii* | PNU-2 |
|  | 19 | *Acinetobacter Baumanii* | PNU-2 |
| 76 | 16 | *Candida tropicalis* | SUTI-1,2 |
|  | 26 | *Acinetobacter Baumanii* | PNU-2 |
| 77 | 16 | *Enterococcus faecium/Trichosporium tumefaciens* | LCBI-1,2 |
| 78 | 17 | not identified | PNU-1 |
| 79 | 17 | *Acinetobacter Baumanii* | PNU-2 |
| 80 | 17 | *Candida glabrata* | SUTI-1,2 |
| 81 | 18 | *Candida tropicalis* | SUTI-1,2 |
|  | 24 | *Acinetobacter Baumanii* | PNU-2 |
| 82 | 19 | *Acinetobacter Baumanii/Staphylococcus aureus* | PNU-2 |
|  | 32 | *Klebsiella pneumoniae* | IAB-1 |
|  | 32 | *Klebsiella pneumoniae* | LCBI -1,2 |
|  | 42 | *Acinetobacter Baumanii* | LCBI -1,2 |
| 83 | 19 | *Acinetobacter Baumanii* | LCBI-1/SUTI-1/PNU-2 |
| 84 | 20 | *Enterococcus faecium* | SUTI-1 |
| 85 | 21 | *Candida glabrata* | PNU-3 |
|  | 42 | *Klebsiella pneumoniae* | SUTI-1,2 |
|  | 28 | *Acinetobacter Baumanii* | PNU-2 |
| 86 | 26 | not identified | PNU-1 |
| 87 | 27 | *Staphylococcus haemolyticus* | LCBI -1 |
| 88 | 28 | not identified | IAB-3 |
| 89 | 31 | *Candida glabrata* | SUTI-1,2 |
|  | 41 | *Enterobacter cloacae/Enterobacter Aerogenes* | SUTI-1,2 |
|  | 68 | *Enterococcus faecium* | SUTI-1,2 |
| 90 | 37 | *Klebsiella pneumoniae* | SUTI-1/PNU-2 |
| 91 | 39 | *Candida albicans/Acinetobacter Baumanii* | SUTI-1,2 |
|  | 69 | *Acinetobacter Baumanii* | PNU-2 |
|  | 97 | *Escherichia coli* | SUTI-1,2 |
| 92 | 47 | *Acinetobacter Baumanii* | PNU-2 |
| ^a^ Diagnosis was according to CDC/NHSN criteria[25]. PNU Pneumonia, LUNG, Other infections of the lower respiratory tract, SUTI Symptomatic urinary tract infection, OUTI Other infections of the urinary tract, GIT Gastrointestinal tract infection, IAB Intraabdominal infection, LCBI Laboratory-confirmed bloodstream infection, DI Disseminated infection, ST Soft tissue infection. The diagnostic criteria was expressed as abbreviation plus criteria number in CDC/NHSN criteria. For example, SUTI-2 stands for this case meets the second diagnostic criteria of symptomatic urinary tract infection. | | | |
