# Supplementary material for: Clinical characteristics, risk factors, immune status and prognosis of secondary infection of sepsis: a retrospective observational study
Source: BMC Anesthesiol. 2019 Oct 18;19:185. doi: 10.1186/s12871-019-0849-9 (PMC6800505; doi:10.1186/s12871-019-0849-9)
Supplement: Supplementary file 3 — Additional file 3: Table S2. Results of the comparison of the change of HLA-DR expression and serum cytokines levels. [file 12871_2019_849_MOESM3_ESM.docx]

| **Table S2. Results of the comparison of the change of HLA-DR expression and serum cytokines levels** | | | | |
| --- | --- | --- | --- | --- |
|  | *P* value (SI/NSI) ^a^ | | |  |
| Biomarkers | day 3 vs. day 1 | day 7 vs. day 3 | day 7 vs. day 1 |  |
| Monocyte HLA-DR expression | 0.843/0.149 | 0.689/0.721 | 0.414/0.984 |  |
| Level of serum cytokines |  |  |  |  |
| IL-6 | **0.023/0.004** | 0.285/0.248 | **<0.001/<0.001** |  |
| IL-8 | 0.984/**<0.001** | 0.08/1 | 0.052**/0.003** |  |
| IL-10 | 0.182/0.995 | 0.07/0.681 | **0.004/**0.376 |  |

^a^ SI secondary infection, NSI non-secondary infection
