# Supplementary material for: Clinical characteristics, risk factors, immune status and prognosis of secondary infection of sepsis: a retrospective observational study
Source: BMC Anesthesiol. 2019 Oct 18;19:185. doi: 10.1186/s12871-019-0849-9 (PMC6800505; doi:10.1186/s12871-019-0849-9)
Supplement: Supplementary file 4 — Additional file 4: Table S3. Characteristics of the septic patients classified according to the prognosis. [file 12871_2019_849_MOESM4_ESM.docx]

| **Table S3. Characteristics of the septic patients classified according to the prognosis** | | | |
| --- | --- | --- | --- |
|  | In-hospital mortality n = 94 | Survival n= 203 | *P* value |
| Baseline characteristics |  |  |  |
| Age, median (25th,75th) | 69 (58-77) | 64 (47-76) | 0.117 |
| > 65 years, n (%) | 59 (62.8) | 96 (47.3) | **0.013** |
| Men, n (%) | 64 (68.1) | 132 (65.0) | 0.605 |
| Comorbidities, n (%) |  |  |  |
| None | 13 (13.8) | 43 (21.2) | 0.132 |
| Hypertension | 40 (42.6) | 84 (41.4) | 0.849 |
| Other cardiovascular diseases ^a^ | 15 (16.0) | 25 (12.3) | 0.392 |
| Diabetes mellitus | 24 (25.5) | 45 (22.2) | 0.523 |
| Cerebrovascular diseases | 9 (9.6) | 10 (4.9) | 0.128 |
| Respiratory diseases | 6 (6.4) | 26 (12.8) | 0.097 |
| Hepatitis and cirrhosis | 2 (2.1) | 11 (5.4) | 0.239 |
| Renal insufficiency | 6 (6.4) | 13 (6.4) | 0.995 |
| Malignancy | 9 (9.6) | 16 (7.9) | 0.625 |
| Immunosuppression | 16 (17) | 20 (9.9) | 0.078 |
| Smoker, n (%) | 37 (39.4) | 64 (31.5) | 0.185 |
| Site of infection, n (%) |  |  |  |
| Respiratory tract | 76 (80.9) | 140 (69.0) | **0.032** |
| Abdomen | 17 (18.1) | 45 (22.2) | 0.421 |
| Urinary tract | 4 (4.3) | 18 (8.9) | 0.158 |
| Skin and soft tissue | 4 (4.3) | 8 (3.9) | 1 |
| Blood stream | 3 (3.2) | 1 (0.5) | 0.095 |
| More than one sites | 9 (9.6) | 12 (5.9) | 0.252 |
| In shock on admission, n (%) | 39 (41.5) | 38 (18.7) | **<0.001** |
| Severity of disease, median (25th,75th) |  |  |  |
| APACHE II score | 19 (14-26) | 11 (6-16) | **<0.001** |
| SOFA score | 6 (4-10.3) | 3 (2-5) | **<0.001** |
| Interventions, n (%) ^b^ |  |  |  |
| Glucocorticoid | 55 (58.5) | 76 (37.4) | **0.001** |
| Anticoagulation therapy | 40 (42.6) | 68 (33.5) | 0.131 |
| Mechanical ventilation | 82 (87.2) | 95 (46.8) | **<0.001** |
| Urinary catheterization | 73 (77.7) | 86 (43.4) | **<0.001** |
| Deep venous catheterization | 67 (71.3) | 77 (37.9) | **<0.001** |
| Continuous renal replacement therapy | 20 (21.3) | 12 (5.9) | **<0.001** |
| Blood transfusion | 33 (35.1) | 27 (13.3) | **<0.001** |
| LOS, median (25th,75th) |  |  |  |
| In-hospital | 9 (5-22) | 15 (10-23) | **0.003** |
| ICU | 9 (4-22) | 14 (9-22) | **0.005** |
| Secondary infection, n (%) | 42 (44.7) | 50 (24.6) | **0.001** |
| ^a^ Other cardiovascular diseases included coronary heart disease, arrhythmia, myocardiosis and valvular heart disease. | | | |
| ^b^ With regard to patients with secondary infection, it referred to the interventions before and after the onset of secondary infection. | | | |
