# Supplementary material for: Clinical characteristics, risk factors, immune status and prognosis of secondary infection of sepsis: a retrospective observational study
Source: BMC Anesthesiol. 2019 Oct 18;19:185. doi: 10.1186/s12871-019-0849-9 (PMC6800505; doi:10.1186/s12871-019-0849-9)
Supplement: Supplementary file 5 — Additional file 5: Table S4. Results of multivariate logistic regression test of risk factors of the in-hospital death. [file 12871_2019_849_MOESM5_ESM.docx]

| **Table S4. Results of multivariate logistic regression test of the risk factors of in-hospital death** | | | | | | |
| --- | --- | --- | --- | --- | --- | --- |
| Variables ^a^ | Partial regression coefficient | Standard error | Wald χ2 | *P* value | OR | 95% CI |
| APACHE II score | 0.084 | 0.030 | 7.907 | **0.005** | 1.087 | 1.026-1.152 |
| SOFA score | 0.119 | 0.071 | 2.756 | 0.097 | 1.126 | 0.979-1.295 |
| Interventions |  |  |  |  |  |  |
| Glucocorticoid | 0.964 | 0.376 | 6.581 | **0.010** | 2.623 | 1.256-5.479 |
| Blood transfusion | 0.949 | 0.463 | 4.199 | **0.040** | 2.582 | 1.042-6.398 |
| Urinary catheterization | 0.863 | 0.405 | 4.536 | **0.019** | 2.370 | 1.071-5.242 |
| ICU LOS | -0.069 | 0.018 | 14.952 | **0.000** | 0.934 | 0.902-0.967 |
| Secondary infection | 1.246 | 0.418 | 8.868 | **0.003** | 3.476 | 1.599-8.219 |
| ^a^ Analysis was conducted using method Backward: Conditional and variable Age > 65 years was removed on step 2, shock on admission on step 3, in-hospital LOS on step 4, deep venous catheterization on step 5, continuous renal replacement therapy on step 6 and mechanical ventilation on step 7. | | | | | | |
